# Service Use for Mental Health Problems in People with Delusional-Like Experiences: A Nationwide Population Based Survey

**DOI:** 10.1371/journal.pone.0071951

**Published:** 2013-08-21

**Authors:** Sukanta Saha, John McGrath, James Scott

**Affiliations:** 1 Queensland Centre for Mental Health Research, The Park - Centre for Mental Health, Wacol, Australia; 2 School of Medicine, University of Queensland, St. Lucia, Australia; 3 Department of Psychiatry, University of Queensland, St. Lucia, Australia; 4 Queensland Brain Institute, University of Queensland, St. Lucia, Australia; 5 The University of Queensland Centre for Clinical Research, Herston, Australia; University of South Florida, United States of America

## Abstract

**Objective:**

Previous population-based studies have found that delusional-like experiences (DLEs) are prevalent in the community, and are associated with a wide range of mental health disorders. The aim of the study was to investigate mental health service use by people with DLEs.

**Methods:**

Subjects were drawn from the Australian National Survey of Mental Health and Wellbeing 2007 of 8 841community residents aged between 16 and 85 years. The Composite International Diagnostic Interview (CIDI) was used to identify DLEs. Service utilization was assessed using a module that elicited information about hospital admissions, consultations with various health professionals, and prescription medication use. This study focussed on service use for mental health problems. We used logistic regression to examine the association, adjusting for potential confounding factors.

**Results:**

Of 8 773 included participants, 8.4% (n = 776) positively endorsed one or more DLEs. With respect to consultations for mental health needs, individuals who endorsed DLEs were more likely to consult health professionals compared with those who did not endorse DLEs. Individuals with DLEs were also more likely to use prescription medicine. When we repeated the main analysis in a subgroup excluding any CIDI diagnosis of mental health disorders the results remained largely unchanged.

**Conclusions:**

DLEs are common in the general population, and individuals with DLEs have an increased rate of accessing services for their mental health needs. Individuals endorsing both DLEs and increased help-seeking may identify a group of vulnerable people who have increased risk of developing psychotic illnesses later in life. This needs closer scrutiny in longitudinal prospective studies.

## Introduction

A systematic review of population-based studies found that hallucinations and delusional-like experiences (DLEs) are common among the general population with prevalence ranging between three and twenty percent [1]. Recent reports show that DLEs are non-specifically associated with mental disorders such as depression, anxiety, and drug and alcohol abuse/dependence [2]–[5]. There are also reports that DLEs are associated with traumatic life events [6], [7], general psychological distress, and suicidal ideation, intent and attempts [8], [9].

Both DLEs and hallucinations have been branded as part of an extended phenotype of psychotic disorder or ‘subclinical psychoses’ which may exist along a continuum of symptom severity represented at its extremity by a skewed distribution of clinically diagnosable psychotic disorders [1], [10]–[12]. There is a body of evidence to suggest that DLEs and hallucinations precede the onset of psychosis [13], however their use as a risk marker for future psychosis has limited utility because of their low predictive values. Accumulating evidence indicates that only a small proportion of people with psychotic-like experiences (PLEs: DLEs and hallucinations) actually go on to develop psychotic disorders. Hanssen et al. [14], in a prospective study, found only 8% of a general population sample with PLEs developed a psychotic disorder within 24 months. However, this rate rose to 21% in individuals who had engaged in *help seeking behaviours*, suggesting that PLEs in individuals seeking mental health care have a higher predictive value for future psychotic illness.

To date outcomes of studies linking service use with DLEs and hallucinations have been inconsistent. Murphy et al. [15] found that those who reported PLEs were more likely to have sought help from health professionals. Based on the Psychosis Screening Questionnaire (PSQ), the study showed that individuals who reported thought control, paranoia or strange experiences were twice as likely to have attended a general practitioner (GP). Contact with other types of health professionals (psychologists, psychiatrists and pharmacists) was not assessed in this study. A longitudinal study from Israel found an association between PLEs and future hospitalisation [13]. Based on 4914 subjects aged 25 to 34 years this study reported that those with psychotic-like symptoms were 4 times more likely to be hospitalised with non-affective psychosis within 5 years after baseline measurement. Despite this increased risk of hospitalisation, the authors suggest that the association might not be clinically useful because of poor predictive values. There is also evidence that psychotic-like experiences associated with distress influence help-seeking [16]. However, a recent Japanese study failed to find any association between PLEs and service use for mental health problems [17]. The study was based on student sample aged between 16 and 30 years from two universities and schools.

In Australia, access to health care is readily available and there are few barriers to help seeking. Usually, primary care physicians (GPs) decide the appropriate care pathway for a patient. Australian citizens can present to a GP with any health problem and their consultation is subsidised by the national health insurance scheme (Medicare). People with a mental health problem who consult their GP may then receive ongoing care by the GP [18], or they may be referred on to specialist treatment usually by a psychologist or psychiatrist [19], [20].

We sought to investigate, and clarify further, the issues surrounding DLEs and service utilization for mental health needs in a large nationally representative population-based sample. This database had advantages compared to previous studies: larger sample size, population sampling, and wider range of professional and community supports surveyed. Furthermore, in addition to assessing consultations with various professionals, we examined use of both over-the-counter and prescription medications and hospital admissions. Based on the existing literature, we predicted that those who reported DLEs were more likely to utilise a broad range of health services for mental health related issues. Furthermore, we predicted that those with DLEs but without a diagnosable mental health problem would also be more likely to utilise health services for mental health related issues.

## Methods

### Participants

Subjects were drawn from the Australian National Survey of Mental Health and Wellbeing 2007 (NSMHWB). Details of the methodology have been published elsewhere [21]. In brief, the NSMHWB was a national face-to-face household survey of community residents aged between 16 and 85 years. Sampling was based on random selection from a stratified, multistage area probability sample of private dwellings. Interviews were carried out by trained interviewers from the Australian Bureau of Statistics from August to December 2007. In total, 8 841 individuals participated in the survey that represents an estimated population of 16 million adults.

### Assessment of DSM-IV Diagnoses and Delusional-like Experiences

A modified version of the World Mental Health Survey Initiative of the Composite International Diagnostic Interview (WMH-CIDI 3.0) was used to generate lifetime DSM-IV based diagnoses of a wide range of common mental health disorders including anxiety disorders, depressive disorders, and alcohol or drug abuse/dependence. In keeping with our previous studies [3], [4], [7]–[9], [22]–[24] for the assessment of DLEs, we used items in Section G designed to screen for possible psychosis. Details of the DLEs are given in Table S1. Briefly, the DLEs component was composed of three ‘screen’ items covering the following features of psychotic disorders: delusions of control, thought interference and passivity (Question 1); delusions of reference and persecution (Question 2); and grandiose delusions (Question 3).

### Assessment of Health Service Use

Health service use was assessed using a module in the WMH-CIDI 3.0. This was a purpose-designed measure developed for use in the Australian context. It focussed on respondents’ lifetime as well as 12 months use of services for mental and other health problems. This study focussed on service use for mental health problems. It elicited information about consultations with various health professionals, hospital admissions and prescription medication use. Health professionals included: GPs, psychiatrists, psychologists, other mental health professionals (including mental health nurses and other health professionals providing specialist mental health services). Other health professionals include specialist doctors, other professionals providing general services and complementary/alternative therapists. The module included questions for lifetime service use such as ‘*Have you ever seen a general practitioner about problems with your mental health?’ or ‘Have you ever seen a psychiatrist for problems with your mental health?’* For this study we used *lifetime* use of services for mental health problems only, to remain consistent with outcome variables.

Similarly, data on hospital admissions and prescription medication use were also collected for mental health problems only, and for lifetime ever (‘*Have you ever been admitted overnight or longer in hospital for problems with your mental health? ’; ‘Did you ever get any prescription medication for problems with your mental health?’*).

In keeping with our previous analyses [3], [4], [7]–[9], [22]–[24], individuals who screened positively for schizophrenia (i.e. respondents who reported ‘Yes’ to the item ‘*Had been told at any time by a psychiatrist that they had schizophrenia*’) were excluded from the analyses (n = 68), leaving a total of 8 773 subjects for this study.

### Data Analysis

For the main analyses, we examined the association between DLEs (i.e. at least one of the G screen or probe items endorsed), and service use including consultation with a general practitioner, psychiatrist, psychologist or any health professional for mental health problems. ‘No contact’ was used as the *reference* group for all variables.

Based on previous research of factors associated with DLEs, in Model 1 we adjusted for sex and age at assessment [6], [25]. Model 2 was adjusted for age and sex together with other demographic variables including marital status, migrant status, employment status and educational status.

To explore the findings under more restrictive conditions we repeated the main analyses in the subgroup who had no lifetime diagnosis of any CIDI-derived DSM-IV mental health disorders (in order to examine the strength of the association between DLEs and help-seeking behaviour in subgroups with *subclinical symptoms* only).

The sample was weighted to adjust for differential probabilities of selection within households, over-sampling of population subgroups, and non-response to match census population distribution on a number of geographic and socio-demographic variables [21]. The initial weights were calibrated against known population estimates. Replicate weight variables were developed using the Jack-knife procedure of replication (i.e., the analysis was repeated after one subject was dropped and then the standard error was derived from the distribution of results from all “minus one” resamples) [26]. Analyses were performed using Proc *Surveylogistic* [27] which is designed to analyse complex survey samples using SAS (version 9.2; Cary, NC: SAS Institute). *Surveyfreq* procedure was used for weighted frequency distribution for the exposer as well as independent variables.

## Results

Of the 8 773 subjects included in the study, 776 (8.4%) positively endorsed one or more DLEs screen items (Table 1). With respect to consultations for mental health needs, individuals who endorsed DLEs were almost three times more likely to have consulted any clinician compared with those who did not endorse DLEs (adjusted OR and 95% CI 2.65; 2.09–3.34). Assistance was sought from psychologists, psychiatrists and general practitioners. When we repeated the main analysis in a subgroup lacking any CIDI diagnosis of mental health disorders, those who endorsed DLEs were almost twice as likely to have consulted a general practitioner or a psychologist for their mental health needs (adjusted ORs and 95% CI 1.88; 1.20–2.93 and 1.89; 1.04–3.44 respectively), but they were not more likely to have seen a psychiatrist.

**Table 1:** Relationship between service utilization for mental health (any consultation) and delusional-like experiences.

Any consultation *Lifetime* for mental health problems)			Delusional-like experiences: full sample (n = 8773)				Delusional-like experiences: in a subgroup lacking any CIDI diagnosis of lifetime mental health disorders (n = 5088)			
			Yes^1^	No^1^	Model 1^2^	Model 2^3^	Yes^1^	No^1^	Model 1^2^	Model 2^3^
			(n = 776) n()%	(n = 7997) n(%)	OR^4^ (95% CI^5^)	OR^4^ (95% CI^5^)	(n = 259) n(%)	(n = 4829) n (%)	OR^4^ (95% CI^5^)	OR^4^ (95% CI^5^)
**General practitioner**										
	**Yes**		310 (35.79)	1566 (19.21)	**2.23 (1.82, 2.72)** *	**2.13 (1.73, 2.63)** *	39 (13.63)	396 (7.78)	**1.84 (1.18, 2.87)** *	**1.88 (1.20, 2.93)** *
	**No**		460 (64.21)	6431 (80.79)	Reference	*Reference*	220 (86.37)	4933 (92.22)	*Reference*	*Reference*
**Any psychiatrist**										
	**Yes**		134 (15.27)	599 (7.13)	**2.33 (1.69, 3.19)** *	**2.13 (1.57, 2.89)** *	11 (2.37)	142 (2.57)	0.93 (0.42, 2.10)	0.93 (0.42, 2.07)
	**No**		642 (84.73)	7398 (92.87)	*Reference*	*Reference*	248 ()97.63	4687 (97.43)	*Reference*	*Reference*
**Any psychologist**										
	**Yes**		145 (20.38)	789 (9.63)	**2.26 (1.69, 2.99)** *	**2.14 (1.59, 2.89)** *	21 (6.39)	175 (3.44)	**1.89 (1.08, 3.34)** *	**1.89 (1.04, 3.44)** *
	**No**		601 (79.62)	7208 (90.37)	*Reference*	*Reference*	238 (93.61)	4654 (96.57)	*Reference*	*Reference*
**Any practitioner** $										
	**Yes**		385 (48.78)	2107 (25.26)	**2.72 (2.17, 3.41)** *	**2.65 (2.09, 3.34)** *	56 (18.44)	625 (11.99)	**1.64 (1.17, 2.29)** *	**1.65 (1.17, 2.32)** *
	**No**		391 (51.22)	5890 (74.75)	*Reference*	*Reference*	203 (81.56)	4204 (88.01	*Reference*	*Reference*

1 = Weighted frequency;

2 Model 1 = Adjusted for age and sex;

3 Model 2 = Adjusted for age and sex, migrant status, marital status, educational status, and employment status;

4 OR = Odds Ratio,

5 CI = 95% Confidence Interval;

* significance: *p*<0.001 (shown in bold);

$ ‘Any practitioner’ refers to general practitioner, psychiatrist, psychologist or other health specialists.

Compared with those who did not endorse DLEs, individuals with DLEs had approximately twice the odds of reporting lifetime use of prescription medication, hospitalisation and taking vitamins and herbal medication for their mental health needs in the previous fortnight (Table 2). However, after excluding participants who had a lifetime CIDI diagnosis of any mental disorder, the association between DLEs endorsement and hospitalisation or taking vitamins and herbal medication for their mental health was no longer significant.

**Table 2:** Relationship between service utilization for mental health (hospital admission/prescription medicine use) and delusional-like experiences (n = 8773).

Any consultation (for mental health problems)			Delusional-like experiences: full sample (n = 8773)				Delusional-like experiences: in a subgroup lacking any CIDI diagnosis of lifetime mental health disorders (n = 5088)				
			Yes^1^	No^1^	Model 1^2^	Model 2^3^	Yes^1^		No^1^	Model 1^2^	Model 2^3^
			(n = 776) n(0%	(n = 7997) n(0%	OR^4^ (95% CI^5^)	OR^4^ (95% CI^5^)	(n = 259) n(0%		(n = 4829) n(0%	OR^4^ (95% CI^5^)	OR^4^ (95% CI^5^)
**Any ** ***lifetime*** ** admission in hospital**											
		**Yes**	61 (6.39)	241 (2.73)	**2.42 (1.62, 361)** *	**2.03 (1.36, 3.04)** *	2 (0.59)	41 (0.77)		0.76 (0.06, 9.77)#	–
		**No**	715 (93.60)	7756 (97.26)	*Reference*	*Reference*	257 (99.41)	4788 (99.23)		*Reference*	
**Any ** ***lifetime*** ** use of prescription medication**											
		**Yes**	255 (28.42)	1257 (15.21)	**2.17 (1.69, 2.80)** *	**2.05 (1.59, 2.66)** *	31 (10.75)	321 (6.13)		**1.86 (1.10, 3.15)** *	**1.86 (1.09, 3.16)** *
		**No**	521 (71.58)	6740 (84.79)	*Reference*	*Reference*	228 (89.25)	4508 (93.87)		*Reference*	*Reference*
**Any vitamin/herbal medications used in ** ***last 2 weeks***											
		**Yes**	143 (20.07)	768 (9.11)	**2.42 (1.71, 3.44)** *	**2.32 (1.61, 3.34)** *	31 (8.40)	322 (6.45)		1.31 (0.80, 2.17)	1.32 (0.79, 2.22)
		**No**	633 (79.93)	7229 (90.89)	*Reference*	*Reference*	234 (91.59)	4507 (93.54)		*Reference*	*Reference*

1 = Weighted frequency;

2 Model 1 = Adjusted for age and sex;

3 Model 2 = Adjusted for age and sex, migrant status, marital status, educational status, and employment status;

# = Unadjusted because of low power;

– = no estimate available because of low power;

4 OR = Odds Ratio,

5 CI = 95% Confidence Interval;

* significance: *p*<0.001 (shown in bold).

## Discussion

In this national Australian survey, those participants who endorse DLEs were significantly more likely to have had contact with primary care physicians, psychologists and other health professionals for their mental health problems. After excluding individuals who had a lifetime CIDI diagnosis of any mental disorder, those who endorsed DLEs were still significantly more likely to have had contact with primary care physicians and psychologists.

Given previous studies showing those with DLEs are more likely to experience a broad range of mental health problems [2]–[4], [8], [9], the increased likelihood of contact with health professionals by people with DLEs is unsurprising. It is also consistent with previous studies in England and Israel which reported that individuals with psychotic-like experiences were more likely to have contact with the health care system [13], [15]. However, those with DLEs who did not have a lifetime CIDI diagnosis of a mental disorder were not more likely to have seen a psychiatrist. It seems that people with DLEs are most likely managed by primary care physicians or psychologists because in the absence of a diagnosable mental illness their symptoms are not severe enough to warrant referral to a psychiatrist.

Although people with DLEs are more likely to use prescription medications for their mental health needs than their non-DLEs counterparts, we were not able to determine what type of medications was prescribed. As DLEs are associated with anxiety, depression and psychological distress, we would expect higher usage of psychotropic medications.

These results enrich our understanding of the clinical significance of DLEs. It has become increasingly apparent that DLEs are non-specific markers of poor health. Studies have shown that those with DLEs are more likely to have drug and alcohol problems [4], psychological distress [9], suicidal ideation and behaviours [8], a history of trauma [7], and physical health problems [22]. They are also more likely to be socio-economically disadvantaged and socially isolated [23], [24]. Many of the social and health factors associated with DLEs are also determinants for ongoing mental and physical health problems. Given the contemporaneous and future morbidity in adults with DLEs [13], [28]–[30], screening for sub-clinical psychotic experiences may enable detection of individuals who require enhanced physical and mental health support [31].

It is worth noting that in spite of the increased risk of morbidity associated with DLEs [22] just over 50% of participants with DLEs had *never attended a health practitioner for management of a mental health problem*. It is unclear from this study if these participants were distressed by their experiences but (a) had chosen to avoid health professionals, or (b) were seeking help but could not access any services (i.e. had an unmet need). Alternatively, individuals with DLEs may have been otherwise healthy and had no need for mental health care. Previous studies have shown that those with sub-clinical psychotic experiences have an increased risk of both psychotic and non- psychotic disorders [11], [14], [29]. It is important to determine if those who have persistent DLEs [32] or are experiencing distress arising from DLEs are accessing mental health care as these individuals are most at risk of future morbidity [13], [15].

The interpretation of this study has several caveats. The results are based on a cross-sectional sample, and thus we do not know the sequence of DLEs versus help-seeking behaviour. We used only three screen items to measure delusional experiences and there were no items for hallucinations although previous studies have shown that DLEs as measured by the CIDI are highly correlated with hallucinations [5], [7], [31]. Because the data were obtained from a household survey, some population groups such as homeless individuals and people living in nursing home, hostels etc. were not surveyed. There are also some limitations regarding contact with health professionals. In the dataset, there was no mention of what specific mental problems respondents had when visiting different health care practitioners (e.g. did they seek help for DLEs or a comorbid mental disorder). It was also not known which medications they were prescribed and for what. Previously, we found an association between physical health and DLEs [22]. In a future study, we plan to examine the association between (a) DLEs with and without comorbid physical conditions versus (b) service utilization for general health needs.

In summary, our results indicate that individuals who had DLEs were more likely to seek help from health professionals for their mental health problems independently of any mental health disorders. It is now very apparent that there is a high level of morbidity associated with DLEs, which in turn leads to increased burden on health service providers. With the increased risk to both mental and physical health that people with DLEs experience, and the cost of the associated health care, there is an unambiguous need for studies to intervene with those who experience DLEs.

## Supporting Information

Table S1 **CIDI Screen items for Psychosis (Delusional-like experiences, DLE).**
(DOCX)
