# Supplementary material for: Service Use for Mental Health Problems in People with Delusional-Like Experiences: A Nationwide Population Based Survey
Source: PLoS One. 2013 Aug 21;8(8):e71951. doi: 10.1371/journal.pone.0071951 (PMC3749219; doi:10.1371/journal.pone.0071951)
Supplement: Table S1 — CIDI Screen items for Psychosis (Delusional-like experiences, DLE). (DOCX) [file pone.0071951.s001.docx]

**Table S1. CIDI Screen items for Psychosis (Delusional-like experiences, DLE)**

| Item G1 (PS1*): |
| --- |
| *Have you ever felt that your thoughts were being directly interfered with or controlled by another person?* |
| **Item G2 (PS2*):** |
| *Have you ever had a feeling that people were too interested in you?* |
| **Item G1 (PS3*):** |
| *Do you ever have any special powers that most people lack?* |

*Item PS4^@^:*

*Has a doctor ever told you that you may have schizophrenia?*

*Screen items (lifetime) with answer (Yes/No): ‘Any screen’ items required ‘Yes’ answers to all three questions.

^@^Sample excluded from the analyses (n=68)
